# Phylogeny and taxonomy of three new *Ctenomyces* (Arthrodermataceae, Onygenales) species from China

**DOI:** 10.3897/mycokeys.47.30740

**Published:** 2019-02-20

**Authors:** Zhi-Yuan Zhang, Yan-Feng Han, Wan-Hao Chen, Zong-Qi Liang

**Affiliations:** 1 Institute of Fungus Resources, Department of Ecology, College of Life Sciences, Guizhou University, Guiyang 550025, Guizhou, China Guizhou University Guiyang China; 2 Department of Microbiology, Guiyang College of Traditional Chinese Medicine, Guiyang 550025, Guizhou, China Guiyang College of Traditional Chinese Medicine Guiyang China

**Keywords:** 3 new species, Filamentous fungi, *Ctenomyces*, Morphology, Multigene

## Abstract

Twelve *Ctenomyces* (Arthrodermataceae, Onygenales) strains were obtained and identified during a survey of keratinophilic fungi in soils from China. We used molecular identification combined with morphological evidence to delimit species, circumscribing five species in the genus. Three new species are herein described: *C.albus***sp. nov.**, *C.obovatus***sp. nov.** and *C.peltricolor***sp. nov.** We also described, illustrated and compared the novel species with related species in the morphology.

## Introduction

The genus *Ctenomyces* belongs in the family Arthrodermataceae in the order Onygenales (Wijayawardene et al. 2018) with *C.serratus* as the type species (Eidam 1880, Wijayawardene et al. 2017). *Trichophytonlacticolor* and *Microsporummentagrophytes* were transferred to *Ctenomyces*, being renamed *C.lacticolor* and *C.mentagrophytes* (Langeron and Milochevitch 1930). Subsequently, ten species (*Trichophytondenticulatum*, *T.equinum*, *T.eriotrephon*, *T.farinulentum*, *T.felineum*, *T.griseum*, *T.persicolor*, T.gypseumvar.radioplicatum, *T.viannai* and *Epidermophytongypseum*) were transferred to the genus (Nannizzi 1934). Thereafter, two new species, *C.bossae* and *C.trichophyticus*, were also described (Milochevitch 1935, Szathmáry 1960). Later studies showed that *C.bossae* was misnamed; *C.trichophyticus* was invalid; *C.felineus* was a synonym of *C.serratus*; *C.persicolor* was transferred to *Nannizzia*, and named as *N.persicolor*; *C.mentagrophytes*, *C.equinus* and *C.eriotrephon* were transferred to *Trichophyton* and named as *T.mentagrophytes*, *T.equinum* and *T.eriotrephon*, respectively; *C.lacticolor* and *C.denticulatus* were transferred to *T.mentagrophytes*; the remaining five species *C.farinulentus*, *C.griseus*, *C.radioplicatus*, *C.viannai* and *C.gypseus* were transferred back to *Trichophyton* and *Epidermophyton*, which were eventually regarded as invalid or unclear (Orr and Huehn 1963, de Hoog et al. 2000, de Hoog et al. 2017). Therefore, *C.serratus* was ultimately regarded as the only valid species within the genus (Orr and Huehn 1963).

The main diagnostic criteria of *Ctenomyces* (*sensu*Oorschot 1980) are that conidia are verrucose, thick-walled, lightly pigmented, commonly with ampulliform swellings and mostly longer than 8 μm. Oorschot (1980) regarded *Ctenomyces* as a sexual morph of *Myceliophthora*. Furthermore, he transferred *Chrysosporiumasperatum* to *Myceliophthora* as *M.vellerea* in a taxonomic revision of *Chrysosporium* and allied genera. *Myceliophthoravellerea* was regarded as a synonym of the asexual morph of *Ctenomycesserratus* (Guarro et al. 1985, Chabasse 1988). Phylogenetic analyses, based on ITS rDNA, demonstrated *Myceliophthoravellerea* to be a synonym of *Ctenomycesserratus* (van den Brink et al. 2012). De Hoog et al. (2017) expanded the breadth and understanding of dermatophytes in Arthrodermatacea based on multi-locus molecular data and *Ctenomycesserratus* is used as the only previously validated species of the genus *Ctenomyces*.

Investigation of keratinophilic fungi has been given more attention in some countries (Anbu et al. 2004, Zarrin and Haghgoo 2011, Shadzi et al. 2002). Many researchers have shown that keratinophilic fungi distribution is closely related to human and animal activity (Sharma and Sharma 2010). Therefore, we conducted a survey of keratinophilic fungi in places with high human activity in Guizhou, Shanxi and Gansu provinces in China and isolated 12 strains. By combining the ITS sequence and a multi-gene phylogeny and the morphological characteristics, we identify and describe three new species and one new record of *Ctenomyces* from China.

## Materials and methods

### Isolates

Twelve *Ctenomyces* strains were obtained from soil samples collected in Guizhou, Shanxi and Gansu province of China using a baiting technique (Vanbreuseghem 1952). Sterile chicken feather and human hair were combined with the soil samples and the samples were placed in sterile Petri dishes, which were moistened with sterile distilled water. The baited soil sample Petri dishes were incubated at 25 °C for 1 month and remoistened as necessary. When fungal growth was observed, those feathers with fungal growth were mixed with 9 ml of sterile water in an Erlenmeyer flask and 1 ml of suspensions were evenly spread on plates containing Sabouraud’s dextrose agar (SDA) with chloramphenicol and cycloheximide medium. The plates were incubated at 25 °C. The pure culture were then transferred to potato dextrose agar (PDA) plates for purification, the isolates were inoculated to test-tube slants and stored at 4 °C.

All holotypes and isotypes were deposited in the Mycological Herbarium of the Institute of Microbiology, Chinese Academy of Sciences, Beijing, China (HMAS). Type strains and ex-type living cultures were deposited in the China General Microbiological Culture Collection Center (CGMCC) and the Institute of Fungus Resources, Guizhou University (GZUIFR). Taxonomic information of the new taxa was deposited in MycoBank (www.MycoBank.Org).

### Morphology

Isolates were transferred to potato dextrose plates, incubated at 25 °C for 14 days and subjected to macroscopic examination. Fungal microscopic features were examined with a Nikon Ti-U microscope (Nikon, Japan) and photographed. Diagnostic features were then illustrated on the basis of these observations. Finally, the fungi were morphologically identified according to colony characteristics, conidiogenous structures and conidia (*sensu*Oorschot 1980).

### DNA extraction, PCR amplification, sequencing

Total genomic DNA was extracted from fresh sporulating cultures after 14 days at 25 °C using a Fungal DNA Mini Kit (Omega Biotech, Doraville, GA, USA) according to the manufacturer’s protocol and then stored at -20 °C. Three regions were amplified and sequenced, including the internal transcribed spacer (ITS) region using primers ITS1 and ITS4 (White et al. 1990); partial fragments of the RNA polymerase II largest subunit 2 (RPB2) gene region using primers 5F-Eur and 7cR-Eur (van den Brink et al. 2012); partial fragments of the translation elongation factor 1-alpha (EF1A) gene region using primers EF1-983F and EF1-2218R (van den Brink et al. 2012). The PCR mixture was prepared using a commercial kit (TSINGKE Biological Technology, Kunming, China) and contained 5 μl 10 × reaction buffer, 0.4 μl dNTPs (25μM), 0.2 μl T6 DNA polymerase (5 U/μl), 1 μl of each primer and 2 μl DNA template in a final volume of 25 μl. Reaction mixtures were pre-heated at 98 °C for 2 min and PCR was performed as follows: 30 cycles of 10 s at 98 °C, 10 s at 55 °C and 10 s 72 °C, with a final extension at 72 °C for 5 min and cooling at 4 °C. The PCR conditions were the same for all three markers. The resulting PCR products were sequenced by TSINGKE Biological Technology (Kunming, China) using the corresponding primers.

### Phylogenetic analysis

Sequence data from the nine genera of Arthrodermataceae and *Myceliophthoralutea* sequences were used in the phylogenetic analysis. Details of newly generated and reference sequences retrieved from GenBank are listed in Table 1. Multiple sequence alignments for ITS, EF1A and RPB2 were achieved with MAFFT v.7.037b (Katoh and Standley 2013) and manually edited in the MEGA 6.06 (Tamura et al. 2013).

**Figure 1.**
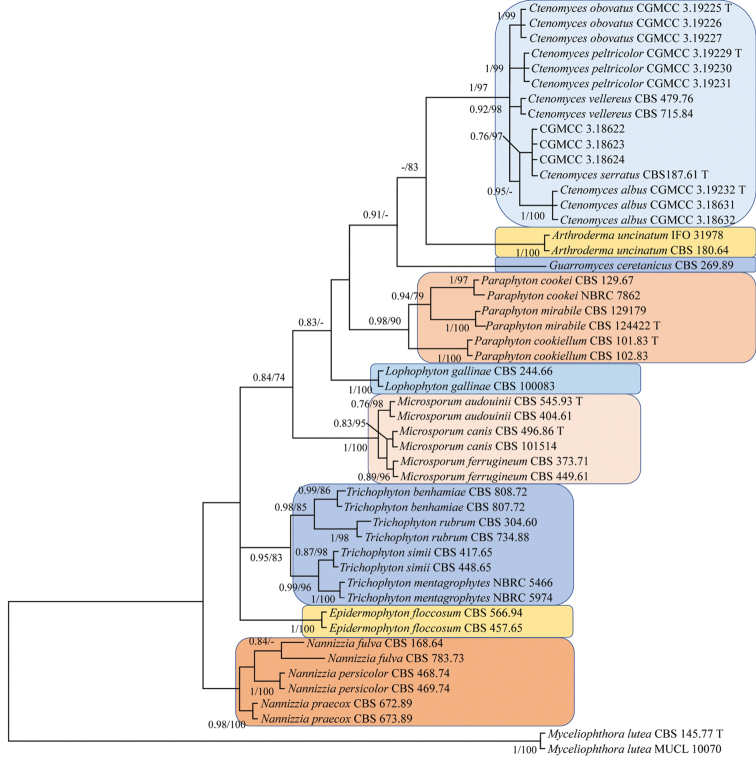
Phylogenetic tree of Arthrodermataceae based on the ITS dataset and *Myceliophthoralutea* (CBS 145.77 and MUCL 10070) as the outgroup taxon. Numbers at nodes are Bayesian posterior probabilities (left, BPP ≥0.75) and maximum likelihood bootstrap values (right, BS ≥70%).

A total of 50 ITS sequences of 23 species and including *Myceliophthoralutea* (CBS 145.77 and MUCL 10070) as the outgroup taxon were used in the analysis. The data were analysed phylogenetically using Bayesian Markov chain Monte Carlo (MCMC) and maximum likelihood (ML). For the Bayesian analysis, two simultaneous Bayesian Inference (BI) Markov chain Monte Carlo runs were also executed for 10,000,000 generations, saving trees every 500 generations. Modeltest v3.7 suggested the GTR+I+G as the best-fit evolutionary model for dataset (Posada and Crandall 1988). After the BI analysis, each run was examined using the programme Tracer v1.5 (Drummond and Rambaut 2007) to determine whether the burn-in period was sufficient and to confirm that both runs had converged. ML analyses were performed using RAXML (Stamatakis and Alachiotis 2010) with the graphical user interface (GUI) (Silvestro and Michalak 2012) implementation and the GTRGAMMA model. The BI and ML analysis trees are available in TreeBASE (http://purl.org/phylo/treebase/phylows/study/TB2:S23736) and a consensus tree is presented in Figure 1.

**Figure 2.**
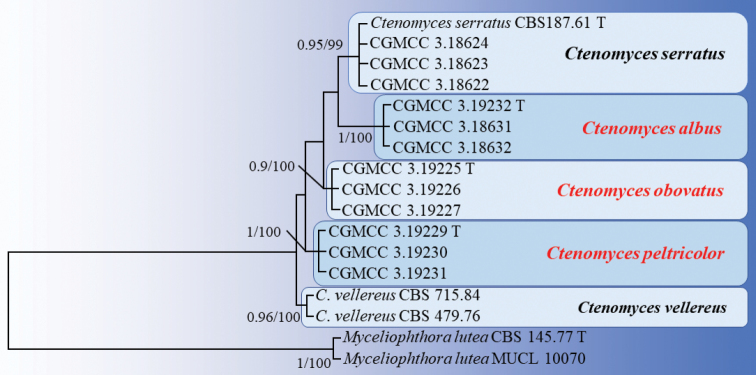
Phylogenetic tree of *Ctenomyces* based on the ITS+EF1A+RPB2 dataset and *Myceliophthoralutea* (CBS 145.77 and MUCL 10070) as the outgroup taxon. Numbers at nodes are Bayesian posterior probabilities (left, BPP ≥0.9) and maximum likelihood bootstrap values (right, ≥95%).

A concatenated dataset (ITS+EF1A+RPB2) of five *Ctenomyces* species and *Myceliophthoralutea* (CBS 145.77 and MUCL 10070) was assembled using SequenceMatrix v. 1.7.8 (Vaidya 2011). Concordance between genes was assessed with the ‘hompart’ command of PAUP4.0b10 (Swofford 2002). Maximum likelihood (ML) phylogenetic analyses of the datasets were performed using RAxML (Stamatakis and Alachiotis 2010) with the graphical user interface (GUI) (Silvestro and Michalak 2012) implementation and the General Time Reversible (GTR) model. Bootstrap analysis with 1,000 replicates was used to estimate nodal support. Two simultaneous Bayesian Inference Markov chain Monte Carlo runs were also executed for 10,000,000 generations, saving trees every 500 generations. Modeltest v3.7 suggested the GTR+I+G as the best-fit evolutionary model for the dataset (Posada and Crandall 1988). After the BI analyses, each run was examined using the programme Tracer v1.5 (Drummond and Rambaut 2007) to determine whether the burn-in period was sufficient and to confirm that both runs had converged. The BI and ML analyses trees are available in TreeBASE (http://purl.org/phylo/treebase/phylows/study/TB2:S23736) and a consensus tree is presented in Figure 2.

## Results

### Phylogeny analysis

The ITS sequence alignment comprised 50 strains of 23 species (Table 1). The final dataset comprised 609 characters after alignment, which included nine genera of Arthrodermataceeae: *Arthroderma*, *Ctenomyces*, *Epidermophyton*, *Guarromyces*, *Lophophyton*, *Microsporum*, *Nannizzia*, *Paraphyton* and *Trichophyton* and *Myceliophthoralutea* (CBS 145.77 and MUCL 10070). No significant differences in topology were observed between the BI and ML phylogenies. The phylogenies show that each genus to cluster into the expected subclades (Figure 1). The *Ctenomyces* strains all cluster in a single clade with good nodal support (BPP 1, BS 97%) and were divided into four subclades, including the *C.vellereus* and the type species, *C.serratus*. All species of *Ctenomyces* clustered in separate well-supported subclades comprising *C.serratus* (BPP 0.76, BS 97%), *C.vellereus* (BPP 0.92, BS 98%), *C.albus* (BPP 1, BS 100%), *C.obovatus* (BPP 1, BS 99%) and *C.peltricolor* (BPP 1, BS 99%).

The combined ITS+EF1A+RPB2 sequence alignment comprised 17 taxa of six species within *Ctenomyces* and *Myceliophthora* (Table 1). The total length of sequences were 1,719 (ITS: 453 bp, EF1A: 728 bp, RPB2: 538 bp) characters and *M.lutea* (CBS 145.77 and MUCL 10070) was the designated outgroup taxon. The BI and ML analysis yielded congruent tree topology (Figure 2). The phylogenies show that each genus was sorted into expected subclades. Particularly, *C.vellereus*CBS 479.76 and CBS 715.84 strains cluster together with strong support values (BPP 0.96, BS 100%) and were separate from *C.serratus* (CBS 187.71, CGMCC 3.18622, CGMCC 3.18623 and CGMCC 3.18624) clustering together with strong support (BPP 0.95, BS 99%). In addition, our proposed three new species: *C.albus*, *C.obovatus* and *C.peltricolor*, had high support as a single subclade 1/100%, 0.9/100% and 1/100%, respectively.

**Table 1.** Strains included in the phylogenetic analysis.

Taxa	Strain	GenBank accession		
		ITS	EF1A	RPB2
*Arthrodermauncinatum*	IFO 31978	JN134092	KM678197	
	CBS 180.64	MH858408	KM678070	
***Ctenomycesalbus***	**CGMCC 3.19232 T = GZUIFR-QL17.10**	**MH793455**	**MH801900**	**MH801914**
	**CGMCC 3.18631 = GZUIFR-QL17.11**	**MH793456**	**MH801901**	**MH801915**
	**CGMCC 3.18632 = GZUIFR-QL17.12**	**MH793457**	**MH801902**	**MH801916**
***C.obovatus***	**CGMCC 3.19225 T = GZUIFR-L15**020	**MH793449**	**MH801894**	**MH801908**
	**CGMCC 3.19226 = GZUIFR-L15021**	**MH793450**	**MH801895**	**MH801909**
	**CGMCC 3.19227 = GZUIFR-L15022**	**MH793451**	**MH801896**	**MH801910**
***C.peltricolor***	**CGMCC 3.19229 T = GZUIFR-C03010**	**MH793458**	**MH801903**	**MH801917**
	**CGMCC 3.19230 = GZUIFR-C03011**	**MH793459**	**MH801904**	**MH801918**
	**CGMCC 3.19231 = GZUIFR-C03012**	**MH793460**	**MH801905**	**MH801919**
*C.serratus*	CBS 187.61 **T**	AJ877222		
	**CGMCC 3.18622 = ZUIFR-S37.1**	**MH793452**	**MH801897**	**MH801911**
	**CGMCC 3.18623 = GZUIFR-S37.2**	**MH793453**	**MH801898**	**MH801912**
	**CGMCC 3.18624 = GZUIFR-S37.3**	**MH793454**	**MH801899**	**MH801913**
*C.vellereus*	CBS 479.76	HQ871797	HQ871749	HQ871840
	CBS 715.84	HQ871795	HQ871747	HQ871841
*Epidermophytonfloccosum*	CBS 566.94	MH862489		
	CBS 457.65	MH858667		
*Guarromycesceretanicus*	CBS 269.89	MF926403		
*Lophophytongallinae*	CBS 244.66	MH858789		
	CBS 100083	MF926355		
*Microsporumaudouinii*	CBS 545.93 **T**	KT155940		
	CBS 404.61	MF926387		
*M.canis*	CBS 496.86 **T**	KT155928		
	CBS 101514	KT155672		
*M.ferrugineum*	CBS 449.61	KT155902		
	CBS 373.71	KT155886		
*Nannizziafulva*	CBS 168.64	MH378229		
	CBS 783.73	MH378230		
*N.persicolor*	CBS 468.74	AJ000615		
	CBS 469.74	AJ000614		
*N.praecox*	CBS 672.89	MH378245		
	CBS 673.89	MH378246	KM678113	
*Paraphytoncookei*	CBS 129.67	MH858923	KM678064	
	NBRC 7862	JN134140	KM678208	
*P.cookiellum*	CBS 101.83 **T**	KT155670		
	CBS 102.83	KT155674		
*P.mirabile*	CBS 129179	MF926385		
	CBS 124422 **T**	MF926384		
*Trichophytonbenhamiae*	CBS 808.72	MH860614	KM678050	
	CBS 807.72	MH860613	KM678118	
*T.mentagrophytes*	NBRC 5466	JN134100	KM678200	
	NBRC 5974	JN134103	KM678206	
*T.rubrum*	CBS 304.60	AJ270807	KM678081	
	CBS 734.88	AJ270800	KM678115	
*T.simii*	CBS 417.65	MH858646	KM678090	
	CBS 448.65	MH858665	KM678099	
*Myceliophthoralutea*	CBS 145.77 **T**	HQ871775	HQ871722	HQ871816
	MUCL 10070	LK932701	LK932710	LK932724

**T**= type strains, strain and sequences generated in this study are shown in **bold.**CBS: Westerdijk Fungal Biodiversity Institute, Utrecht, The Netherlands; CGMCC: China General Microbiological Culture Collection; NBRC: NITE Biological Resource Centre, Japan; IFO: Institute for Fermentation, Osaka, Yodogawa-ku, Osaka, Japan; GZUIFR: Guizhou University, Institute of Fungus Resources; MUCL: Belgian Co-ordinated Collections of Micro-organisms, Belgium.

## Taxonomy

### Ctenomyces albus

Taxon classificationFungiOnygenalesArthrodermataceae

Y.F. Han, Z.Q. Liang & Z.Y. Zhang sp. nov.

827872

Figure 3

#### Holotype.

CHINA, Guizhou Province, on soil, Sept. 2016, Z.Y. Zhang (HMAS 255389, holotype, ex-type culture CGMCC 3.19232).

#### Paratypes.

CHINA, Guizhou Province, on soil, Sept. 2016, Z.Y. Zhang, dried cultures HMAS 255442 and HMAS 255443, isolates CGMCC 3.18631 (GZUIFR-QL17.11) and CGMCC 3.18632 (GZUIFR-QL17.12).

#### Etymology.

Referring to the white colony.

**Figure 3.**
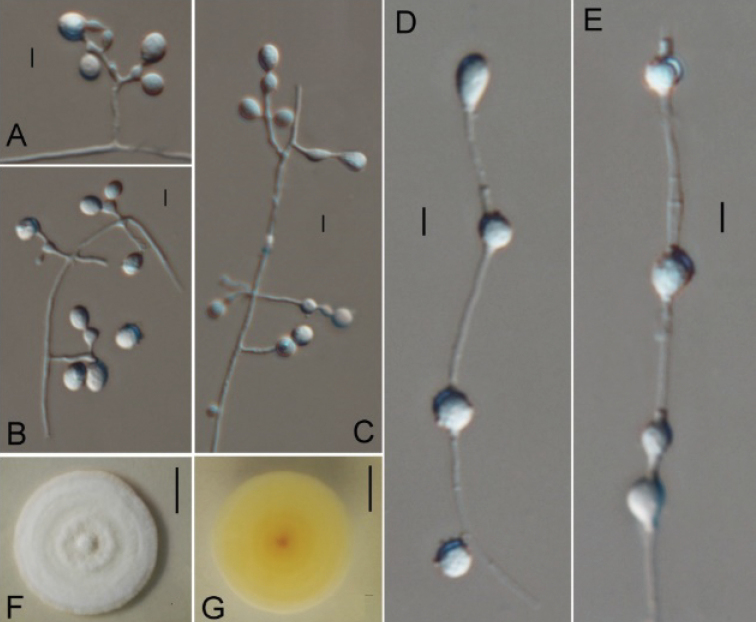
*Ctenomycesalbus* (from ex-holotype strain CGMCC 3.19232). **A–C** Conidiogenous structures and conidia **D, E** Intercalary conidia **F, G** Colony on PDA at day 14. Scale bars: 10 µm (**A–E**); 10 mm (**F, G**).

#### Description.

Aerial hyphae hyaline, smooth, septate, branched, 1.1–2.4 μm wide; racquet hyphae absent. Terminal and lateral conidia borne on hyphae, short protrusions, side branches or an ampulliform swelling. Conidia solitary or in series of up to 2–3 conidia connected by short and slim hypha, ellipsoid, smooth- or rough-walled, verrucose, 12.8–18.6 × 10.8–14.7 μm (*x*‒ = 15.4 × 12.5 μm, n=15). Intercalary conidia present, subglobose or ellipsoidal, smooth- or rough-walled, 13.1–16.9 × 11.2–14.4 μm (*x*‒ = 14.5 × 12.6 μm, n=15).

#### Culture characteristics.

Colonies on PDA growing in the dark reaching 32 mm diam. in 14 d at 25 °C, white, short fluffy to powdery, appearing some annulations, rounded, margin regular and defined. Reverse yellowish.

#### Notes.

*Ctenomycesalbus* is distinct from other species as it is the only species with intercalary conidia in the genus. In addition, our ITS and polygenic phylogeny showed that three isolates of *C.albus* were in a clade sister to *C.serratus* (Figures 1, 2) and clearly separate from other species. Following Jeewon and Hyde’s (2016) guidelines on new species delimitation, there were 37 bp (base pair) differences amongst 508 nucleotides ITS sequences between the isolate CGMCC 3.19232 and *C.serratus*CBS 187.61 (only ITS sequence data are available, EF1A and RPB2 are lacking), which also supports them as distinct different species. Therefore, we introduce *C.albus* sp. nov. in this study.

### Ctenomyces obovatus

Taxon classificationFungiOnygenalesArthrodermataceae

Y.F. Han, Z.Q. Liang & Z.Y. Zhang sp. nov.

827869

Figure 4

#### Holotype.

CHINA, Shanxi Province, on soil, Nov. 2017, Z.Y. Zhang (HMAS 255446, holotype, ex-type culture CGMCC 3.19225).

#### Paratypes.

CHINA, Shanxi Province, on soil, Nov. 2017, Z.Y. Zhang, dried cultures HMAS 255447 and HMAS 255448, isolates CGMCC 3.19226 (GZUIFR-L15021) and CGMCC 3.19227 (GZUIFR-L15023).

#### Etymology.

Referring to the obovoid conidia.

**Figure 4.**
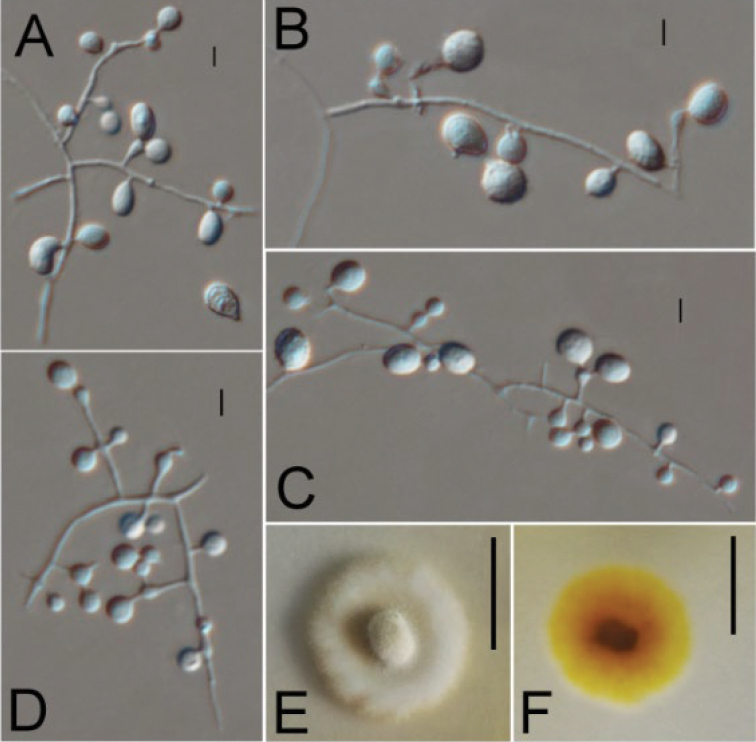
*Ctenomycesobovatus* (from ex-holotype strain CGMCC 3.19225). **A–D** Conidiogenous structures and conidia **E, F** Colony on PDA at day 14. Scale bars: 10 µm (**A–D**); 10 mm (**E, F**).

#### Description.

Aerial hyphae hyaline, smooth, septate, abundant branched, 1.2–2.4 μm wide; racquet hyphae absent. Terminal and lateral conidia borne on hyphae, short protrusions, side branches or an ampulliform swelling. Conidia solitary or in series of up to 2 conidia, ellipsoidal, obovoid, smooth- or rough-walled, verrucose, spinate, 10.3–17.3 × 9.7–10.5 μm (*x*‒ = 14.5 × 10 μm, n=15). Intercalary conidia absent.

#### Culture characteristics.

Colonies on PDA growing in the dark reaching 14–15 mm diam. in 14 d at 25 °C, yellowish, white in the margin; fluffy; rounded, margin regular. Reverse brown.

#### Notes.

*Ctenomycesobovatus* resembles *C.vellereus* in conidia size and conidiogenous cells. However, *C.obovatus* is the only species that produces obovoid conidia in this genus. Furthermore, our ITS and multigene phylogeny shows that three isolates of *C.obovatus* formed a single clade separate from other species (Figure 1), which indicates that *C.obovatus* is a new species.

### Ctenomyces peltricolor

Taxon classificationFungiOnygenalesArthrodermataceae

Y.F. Han, Z.Q. Liang & Z.Y. Zhang sp. nov.

827873

Figure 5

#### Holotype.

CHINA, Gansu Province, on soil, Nov. 2017, Z.Y. Zhang (HMAS 255387, holotype, ex-type culture CGMCC 3.19229).

#### Paratypes.

CHINA, Gansu Province, on soil, Nov. 2017, Z.Y. Zhang, dried cultures HMAS 255439 and HMAS 255440, isolates CGMCC 3.19230 (GZUIFR-C03011) and CGMCC 3.19231 (GZUIFR-C03012).

#### Etymology.

Referring to the pewter colony.

**Figure 5.**
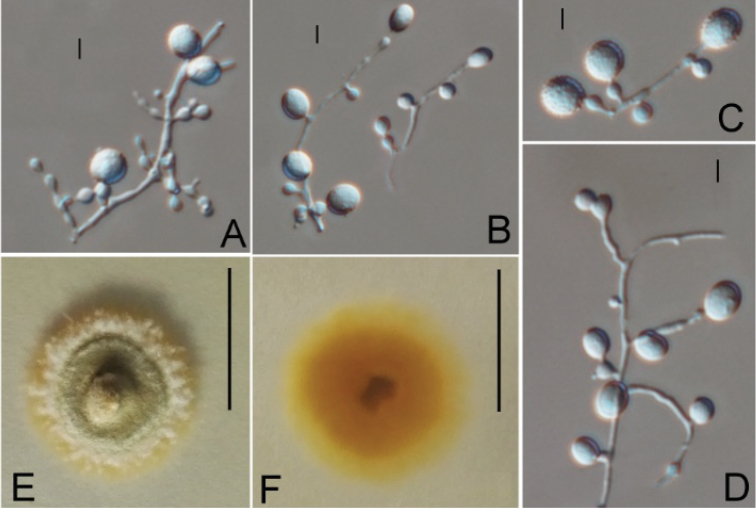
*Ctenomycespeltricolor* (from ex-holotype strain CGMCC 3.19229) **A–D** Conidiogenous structures and conidia **E, F** Colony on PDA at day 14. Scale bars: 10 µm (**A–D**); 10 mm (**E, F**).

#### Description.

Aerial hyphae hyaline, smooth, septate, branched, 1.2–3.3 μm wide; racquet hyphae absent. Terminal and lateral conidia borne on hyphae, short protrusions, side branches or an ampulliform swelling. Conidia solitary, usually only 1 borne on ampulliform swellings; subglobose or globose; smooth- or rough-walled, verrucose, spinate, 8.3–20.2 μm (*x*‒ = 15.5 μm, n=15). Intercalary conidia absent.

#### Culture characteristics.

Colonies on PDA growing in the dark reaching 12 mm diam. in 14 d at 25 °C, pewter at the centre, white in the margin; powdery to floccose at the centre, fluffy in the margin; appearing a circle of annulation; rounded, margin regular. Reverse brown at the centre, yellowish in the margin.

#### Notes.

*Ctenomycespeltricolor* is distinct from other species in its single conidia borne on ampulliform swellings and colony colour. Phylogenetically, the ITS-based phylogenetic analysis (Figure 1) showed that three isolates of *C.peltricolor* cluster in a single clade (BPP 1, BS 100%), consistent with the results of multigene phylogenetic analysis (BPP 1, BS 100%) (Figure 2). Therefore, based on both morphological and phylogenetic evidence, *C.peltricolor* was identified as a new species of *Ctenomyces*.

### Ctenomyces serratus

Taxon classificationFungiOnygenalesArthrodermataceae

Eidam, in Beitrag zur Kenntnis der Gymnoasceen, Beiträge zur Biologie der Pflanzen 3: 267–305 (1880)

827871

Figure 6

#### Description.

Aerial hyphae hyaline, smooth, septate, branched, 0.9–3.3 μm wide; racquet hyphae absent. Terminal and lateral conidia borne on hyphae, short protrusions, side branches or an ampulliform swelling; ampulliform swelling solitary or 2 in series. Conidia solitary or in series of up to 2–3 conidia connected by short and slim hypha, mostly ellipsoidal, sometimes subglobose; smooth- or rough-walled, verrucose, spinate, 11.5–21.9 × 8–15.2 μm (*x*‒ = 18.5 × 13.2 μm, n=15). Intercalary conidia absent.

#### Culture characteristics.

Colonies on PDA growing in the dark reaching 30 mm diam. in 14 d at 25 °C, brown, white in the margin, floccose at the centre, short fluffy in other part, appearing obvious annulation; rounded, margin regular and defined. Reverse yellowish.

#### Specimens examined.

CHINA, Guizhou Province, on soil, Sept. 2016, Z.Y. Zhang, dried cultures HMAS 255390, HMAS 255444 and HMAS 255445, isolates CGMCC 3.18622 (GZUIFR-S37.1), CGMCC 3.18623 (GZUIFR-S37.2) and CGMCC 3.18624 (GZUIFR-S37.3).

#### Known distribution.

Currently known from Australia, England, India, Argentina, Germany (*sensu*Oorschot 1980) and China (this study).

**Figure 6.**
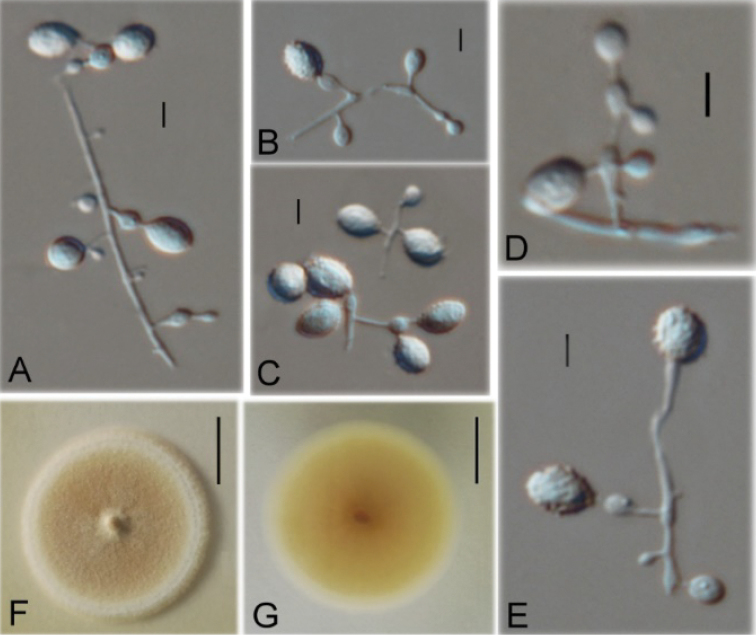
*Ctenomycesserratus* (from strain CGMCC 3.18622) **A–E** Conidiogenous structures and conidia **F, G** Colony on PDA at day 14. Scale bars: 10 µm (**A–E**); 10 mm (**F, G**).

#### Notes.

The Australian collection of *C.serratus* (CBS 187.61) lacks racquet hyphae, conidia occur occasionally on short protrusions and usually 1–2 are borne on ampulliform swellings, solitary or in series of up to 3 conidia separated by short, narrow hyphal segments, initially subhyaline, thin- and smooth-walled, soon becoming reddish-brown, verrucose and thick-walled, ellipsoid, 5–23 × 3.5–12 µm, mature conidia usually 12–23 × 10.5–12 µm, with narrow basal scars (approx. 1 µm) (*sensu*Oorschot 1980). The characteristic features data from the China collections matched rather well with the original description of *C.serratus* reported from Australia. Phylogenetically, our isolates CGMCC 3.18622, CGMCC 3.18623 and CGMCC 3.18624 shared a close relationship with *C.serratus* (Figures 1, 2). Therefore, the isolates CGMCC 3.18622, CGMCC 3.18623 and CGMCC 3.18624 were identified as *C.serratus* which was new to China.

## Discussion

Members of the family Arthrodermataceae (Onygenales) were common in nature, mostly found as saprobes in soil on keratin-rich substrates or associated with vertebrate causing dermatophytosis and other infections. The widely accepted morphology-based taxonomy of dermatophytes in the genera *Trichophyton*, *Microsporum* and *Epidermophyton* was established by Emmons (Emmons 1934). There are three common ecological groups, anthropophilic, zoophilic or geophilic (de Hoog et al. 2017). *Trichophyton* and *Epidermophyton* usually belonged to anthropophiles or zoophilic taxa, *Microsporum* were considered to zoophilic, *Arthroderma* was geophilic. Some species cannot be clearly attributed to one of these groups due to insufficient data. Geophilic dermatophytes have their reservoir in the soil around burrows of specific terrestrial mammals, feeding on keratinous debris. Hence, the difference between geophilic and zoophilic dermatophytes is not always well-resolved. The genus of *Ctenomyces* is usually saprobic or closely related to keratin-rich substrates (Wijayawardene et al. 2017, Deshmukh et al. 2018). In addition, these strains of our study were isolated by using hair and feathers as baiting material. Therefore, we infer the genus *Ctenomyces* to be geophilic and/or zoophilic.

Phylogenetic studies based on the ITS (Graser et al. 2008), partial LSU, the ribosomal 60S protein, partial β-tubulin and translation elongation factor 3 for Arthrodermataceae have shown that the genus *Trichophyton* was polyphyletic and resulted in establishing nine genera, i.e. *Arthroderma*, *Ctenomyces*, *Epidermophyton*, *Guarromyces*, *Lophophyton*, *Microsporum*, *Nannizzia*, *Paraphyton* and *Trichophyton* and it suggested that ITS was the optimal barcoding marker (de Hoog et al. 2017). Therefore, we selected the ITS sequences for phylogenetic analysis of Arthrodermataceae in this study and our results are consistent with the previous studies (de Hoog et al. 2017). *Ctenomycesvellereus* (strains CBS 479.76 and CBS 715.84) had ITS, EF1A and RPB2 sequence data. Hence, we selected the ITS, EF1A and RPB2 genes for phylogenetic analysis of *Ctenomyces*. The results show that the ITS-based and multigene-based phylogenetic analyses have similar results. Although our study revealed that *Ctenomyces* was closely related to *Arthroderma* in Arthrodermataceae, Onygenales, the species of *Ctenomyces* nearly all produce ampulliform swellings, a feature absent in *Arthroderma*.

Guarro et al. (1985), Chabasse (1988) and van den Brink et al. (2012) proposed that *C.vellereus* was a synonym of *C.serratus*, however, these two species have several different characters, the conidia of *C.serratus* are ellipsoid, 12–23 × 10.5–12 µm, while those of *C.vellereus* are subglobose, pyriform or ellipsoid, 4–13 × 3–9 μm (*sensu*Oorschot 1980). Van den Brink et al. (2012) conducted phylogenetic analysis of ITS sequences including only one sequence of *C.serratus* and three sequences of *C.vellereus*. This study used more ITS sequences in *Ctenomyces* for phylogenetic analysis and multigenic phylogeny analysis showed that they were two different species. In our phylogenetic tree, *C.albus*, *C.obovatus*, *C.peltricolor*, *C.serratus* and *C.vellereus* were grouped in five clear clades with good supported value and they are distinct from each other. Thus, based on the present molecular phylogeny, derived from nuclear and ribosomal DNA sequence data, together with morphological evidence, three distinct new *Ctenomyces* species, *C.albus*, *C.obovatus*, *C.peltricolor* and one new record, *C.serratus*, were proposed.

### Key to the species of the genus *Ctenomyces*

**Table:** 

1	Intercalary conidia absen	**2**
–	Intercalary conidia present, subglobose or ellipsoidal	***C.albus***
2	Mostly 1–2 conidia borne on ampulliform swellings	**3**
–	Usually only 1 conidia borne on ampulliform swellings	***C.peltricolor***
3	Conidia less than 20 μm long	**4**
–	Conidia more than 20 μm long	***C.serratus***
4	Conidia obovoid or ellipsoidal	***C.obovatus***
–	Conidia subglobose, pyriform or ellipsoid	***C.vellereus***

## Supplementary Material

XML Treatment for Ctenomyces albus

XML Treatment for Ctenomyces obovatus

XML Treatment for Ctenomyces peltricolor

XML Treatment for Ctenomyces serratus
